# The Development of Immune Thrombocytopenia Due to COVID-19 Presenting as Menorrhagia

**DOI:** 10.7759/cureus.24160

**Published:** 2022-04-15

**Authors:** Sasmith R Menakuru, Adelina Priscu, Vijaypal S Dhillon, Ahmed Salih

**Affiliations:** 1 Internal Medicine, Indiana University Health Ball Memorial Hospital, Muncie, USA

**Keywords:** heavy menstrual bleeding, itp, menorrhagia, covid-19, immune thrombocytopenia

## Abstract

Immune thrombocytopenia (ITP), also known as immune thrombocytopenic purpura, is a hematological disorder characterized by a decreased platelet count, predisposing patients to bleeding. Coronavirus disease 2019 (COVID-19) has been linked to multiple cases of newly diagnosed ITP and is usually found in moderate-to-severe infections, peaking in children and elderly adults. Menorrhagia is the medical term for menstrual periods with abnormally heavy or prolonged bleeding occurring at regular intervals or prolonged uterine bleeding lasting more than seven days. Here, we report the case of a 23-year-old African American female who presented with the chief complaint of menorrhagia and was subsequently diagnosed as having ITP induced by an asymptomatic COVID-19 infection.

## Introduction

Coronavirus disease 2019 (COVID-19), caused by the severe acute respiratory syndrome coronavirus 2 (SARS-CoV-2), has evolved to manifest in various ways. Immune thrombocytopenia (ITP) is one such clinically significant complication that has emerged. ITP is defined as a platelet count below 100,000/µL and is classically associated with bleeding manifestations, such as petechiae [1]. However, the presentation of ITP is varied and can range from mild mucosal bleeding to severe life-threatening hemorrhage. ITP has been associated with several viral illnesses such as Zika, human immunodeficiency virus (HIV), and cytomegalovirus (CMV) [2]. The diagnosis of COVID-19 causing ITP is difficult for clinicians to ascertain due to the various treatments and symptoms associated with the virus, such as using heparin for thromboprophylaxis, concurrent sepsis, disseminated intravascular coagulation, and antibiotic use [3]. It is believed that cytokine release, thrombotic destruction, and autoimmune causes may lead to the development of ITP in COVID-19 patients [4]. Treatment of ITP includes intravenous immunoglobulin (IVIG), glucocorticoids, or thrombopoietin receptor agonists such as eltrombopag in monotherapy or in combination [3].

## Case presentation

A 23-year-old woman presented to an outpatient medical clinic for heavy and prolonged menstrual bleeding. She reported that nothing of this nature had ever happened before, and because it occurred for the past two cycles, she was concerned something was wrong as she felt fatigued and noticed tiny spots on her skin. She also reported that she would be “gushing out blood” and would fill about five pads daily for around nine days and that the bleeding was consistently heavy during this time. She said that she currently had two days of heavy menstrual bleeding, for which she became concerned, so she made an urgent appointment with her primary care provider. She did not present to the clinic after the first cycle because she thought the heavy bleeding would resolve on its own. In addition, she reported noticing very minute spots on her skin that appeared the day before presenting to the clinic; however, she did not think it was serious when compared to her excessive menstrual bleeding. She denied any significant changes in her life and stated that she had a healthy lifestyle with a nutritious diet, little to no stress, and exercised daily. She did not take any medications, including herbal supplements, was not a smoker, and did not drink alcohol or use illicit drugs.

She did not have any family history of bleeding disorders and had never experienced any symptoms related to excessive bleeding before. When asked if she recently had any sick contacts, she said that her boyfriend was diagnosed with COVID-19 40 days ago and was intubated in the hospital. She said that she had no symptoms suggestive of COVID-19 and did not think a test was warranted. She was not vaccinated.

Given her symptomatology, exposure to COVID-19, petechiae, and fatigue, she was sent to the emergency department for a full workup. Complete blood count with differential, complete metabolic panel, abdominal and pelvic computed tomography (CT) scan, chest radiograph, electrocardiogram (EKG), COVID-19 nasopharyngeal swab, blood cultures, D-dimer, lactate dehydrogenase (LDH), an inhibitor of ADAMTS13 and ADAMTS13, ferritin, B-12, folate, international normalized ratio (INR), activated partial thromboplastin time (aPTT), prothrombin time (PT), and fibrinogen were ordered (Table 1). The COVID-19 swab returned positive, although the patient was asymptomatic. CT, EKG, and chest radiograph returned within normal limits. Her previous hemoglobin the year prior was 11.7 g/dL, and her previous platelets were 214 k/mm^3^. Ferritin, B12, and folate were within normal limits. The ADAMTS13 and ADMASTS13 inhibitor returned negative, and blood cultures remained negative throughout hospitalization. Autoimmune causes were ruled out by a negative antinuclear antibody (ANA), antiphospholipid antibody (APLA), antineutrophil cytoplasmic antibody (ANCA), rheumatoid factor (RF), and a direct antiglobulin test. Testing for HIV and hepatitis was negative as well.

**Table 1:** Laboratory values.

Laboratory test	Patient’s value	Normal value
Platelet count	13 k/mm^3^	150–450 k/mm^3^
Hemoglobin	9.6 g/dL	12–15 g/dL
White blood cell count	9.1 k/µL	3.6–10.6 k/µL
Lactate dehydrogenase	319 U/L	140–271 U/L
Fibrinogen	589 mg/dL	200–400 mg/dL
International normalized ratio	1.0	<1.1
Activated partial thromboplastin time	28 seconds	21–35 seconds
Prothrombin time	11.7 seconds	11–13.5 seconds

Due to a high suspicion of ITP, hematology was consulted. A direct monoclonal antibody immobilization of platelet antigen (MAIPA) assay was ordered. The MAIPA assay evaluates the presence of platelet autoantibodies on glycoproteins (GP) Ib/IX, IIb/IIIa, and V. The assay returned positive, and the patient was subsequently diagnosed with ITP due to COVID-19 causing menorrhagia and petechiae. Treatment was started with pulses of dexamethasone 40 mg for four days and 1 g/kg of IVIG for three days simultaneously. She did not develop any signs or symptoms of respiratory infection from COVID-19. Treatment was mainly supportive, and the patient’s platelet counts increased steadily. After seven days, she was discharged from the hospital once heavy menstrual bleeding ceased and platelet counts were above 50 k/mm^3^. She followed up with hematology two weeks and a month after discharge, during which platelet counts were 78 k/mm^3^ and 170 k/mm^3^, respectively. She did not experience any bleeding after discharge and her petechiae resolved.

## Discussion

The hematologic manifestations of COVID-19 are becoming a more pressing issue as the pandemic continues. COVID-19 can unmask a multitude of clinical symptoms, and bleeding diathesis from ITP is one such complication. Thrombocytopenia in any patient with a diagnosis of COVID-19 should be worked up, and clinicians should be aware of the possibility of the development of ITP in COVID-19 patients. The pathogenesis of ITP formation is believed to be due to various mechanisms, including molecular mimicry, underlying immune dysregulation, host generation of antiplatelet antibodies, *suppressor of cytokine signaling 1* gene mutations, cryptic antigen expression, and epitope spread [5]. The prevailing theories include molecular mimicry between viral components and platelet glycoproteins which is also seen in varicella-zoster, HIV, hepatitis C, and CMV [6].

Although the presentation of ITP in COVID-19 has been reported more in elderly patients, it has also been observed in children, with the majority of patients having moderate-to-severe disease [3]. However, as in our patient, symptoms of ITP have also been reported in asymptomatic COVID-19 infections, highlighting the need to test individuals with new-onset thrombocytopenia for COVID-19. In a review of 45 case reports of ITP caused by COVID-19 by Bhattacharjee et al., 38 patients had severe thrombocytopenia, with 28 having severe bleeding. However, 10 patients out of the 38 had no bleeding, even though they had severe thrombocytopenia [3]. The onset of ITP in patients was found to be more common in the second and third weeks of infection with COVID-19 but is likely due to a delay in presentation to either the clinic or hospital [7].

The diagnosis of ITP is a diagnosis of exclusion of other possible causes of thrombocytopenia by performing a series of tests if clinical suspicion is high. In a majority of patients, a platelet count of <100 × 10^9^/L, a low platelet count nadir of <20 × 10^9^/L, a platelet count responsive to therapy (corticosteroids, IVIG, or treatment of the secondary cause), and a positive anti-platelet autoantibody test are enough to confirm the diagnosis of ITP [8]. In difficult cases, testing should first rule out disseminated intravascular coagulation, sepsis, and thrombotic thrombocytopenic purpura by determining D-dimer, fibrinogen, PT, APTT, INR, complement levels, blood smear, and ADAMTS-13. If heparin, antibiotics, or any other drugs known to cause ITP are administered, these should be stopped and worked up as a possible reason for developing thrombocytopenia. Infectious agents such as HIV, varicella-zoster virus, hepatitis C virus, and Epstein-Barr virus should also be ruled out. Autoimmune workup should also be obtained by ordering direct antiglobulin, ANA, ANCA, APLA, and RF. MAIPA can also be performed, which, if positive, can support the diagnosis of ITP [9]. Bone marrow aspiration is usually not necessary unless there are abnormalities in the peripheral blood smear. In patients with decreased platelet counts and symptoms of ITP, COVID-19 must be ruled out.

The goal of treatment for patients with ITP is to prevent severe bleeding, steadily increase platelet count, and provide supportive care. The most commonly used treatments include IVIG and glucocorticoids as first-line and thrombopoietin receptor agonists (TP-RA) as second-line. There have been reports of patients with ITP with a platelet count greater than 30,000/µL and minor mucosal bleeding resolving spontaneously [10]. The American Society of Hematology recommends dexamethasone 40 mg/day for four days or prednisone 1 mg/kg/day for up to six weeks, depending on the response to ITP treatment [11]. IVIG 400 mg/kg/day or 1 g/kg for one to three days is appropriate for patients at risk of severe bleeding as it produces a rapid increase in platelet count in 12-24 hours [12]. TP-RA can also be used as it increases platelet counts one to two weeks after administration and helps prevent recurrence; however, it is known to cause hepatotoxicity and thrombotic complications and should be reserved as a second-line agent [12].

## Conclusions

Although ITP has been associated with COVID-19, its presentation in asymptomatic young adults as heavy prolonged menstrual bleeding has not been reported in the current literature, highlighting the uniqueness of this case. We believe that patients presenting with signs and symptoms of ITP should be worked up for COVID, especially if there is a history of exposure. Prompt diagnosis by excluding other pathologies and treatment with IVIG and glucocorticoids is necessary if platelet counts are abnormal, allowing for a positive outcome.
